# Intelligent Defect Diagnosis of Rolling Element Bearings under Variable Operating Conditions Using Convolutional Neural Network and Order Maps

**DOI:** 10.3390/s22052026

**Published:** 2022-03-04

**Authors:** Syed Muhammad Tayyab, Steven Chatterton, Paolo Pennacchi

**Affiliations:** Department of Mechanical Engineering, Politecnico di Milano, Via G. La Masa 1, 20156 Milan, Italy; syedmuhammad.tayyab@polimi.it (S.M.T.); paolo.pennacchi@polimi.it (P.P.)

**Keywords:** rolling element bearings, intelligent defect diagnosis, artificial intelligence, convolutional neural network, variable operating conditions, order maps

## Abstract

Vibration analysis is an established method for fault detection and diagnosis of rolling element bearings. However, it is an expert oriented exercise. To relieve the experts, the use of Artificial Intelligence (AI) techniques such as deep neural networks, especially convolutional neural networks (CNN) have gained the attention of researchers because of their image classification and recognition capability. Most researchers convert the vibration signal into representative time frequency vibration images such as spectrograms and scalograms. These images are used as inputs to train the CNN model for fault diagnosis. Commonly, fault diagnosis is performed under same operating conditions, where models are trained and deployed for prediction under the same operating conditions. However, outside the laboratory environment, in real world applications, different operating conditions, such as variable speed, may be encountered. With the change in speed, the characteristic frequencies of the vibration signal will also change, which will result in changing the vibration image. Consequently, the performance of the CNN model may drop significantly for prediction under different operating conditions. Accessing the training data from all potential operating conditions may not be feasible for most real-world applications. Therefore, there is a need to find some signal properties which are invariant to change in operating conditions and only change due to change in health state so that models trained under one set of operating conditions may predict correctly under different operating conditions. This paper proposes a defect diagnosis method for rolling element bearings, under variable operating conditions (speed and load) based on CNN and order maps. These maps exhibit consistent properties under varying speed; therefore, they can be used to train the CNN model for fault diagnosis under variable speed. The effect of load change on these order maps is experimentally studied and it is found that the proposed method can undertake fault diagnosis on rolling element bearings under variable speeds and loads with high accuracy.

## 1. Introduction

Rolling element bearings are a vital part of rotating machines as they support the shaft, take load, and reduce the friction between different components. Their health status has a significant effect on the performance and availability of industrial machinery. As they are one of the most vulnerable components of rotating machines, bearing faults are a major cause of machine defects [1]. Bearing failure may cause serious damage to a machine and may result in the unavailability of important machinery, leading to financial loss and serious safety hazards. Therefore, the early defect diagnosis of rolling element bearings is crucial for the uninterrupted availability and operation of machines. To manage the potential failure of machinery, accurate fault detection and the diagnosis of bearings has gained significant researcher attention. Different methods have been introduced by researchers defectto diagnose the s of rolling element bearings, though vibration signal analysis in the time domain, frequency domain and time frequency domain have been most widely used [2,3]. However, it is an expert oriented task, and human involvement in general is not very effective or efficient in terms of responding quickly to large volumes of data. To minimize human dependency, intelligent condition monitoring techniques are attracting the attention of researchers. Over the past two decades, researchers have proposed many new approaches related to artificial intelligence techniques or conventional machine learning (ML) techniques [4], such as, the use of artificial neural networks (ANN) for rolling element bearing fault diagnosis [5,6], the principal omponent analysis (PCA) -based feature selection scheme for machine defect classification [7], a support vector machine (SVM) -based approach for machine condition monitoring and fault diagnosis [8], a bearing defect diagnosis approach, based on ANN and SVM [9,10], a hierarchical diagnosis network approach for fault pattern recognition in rolling element bearings [11], and the intelligent defect diagnostics of bearings and gears using k-nearest neighbors (KNN) as classifier along with the genetic algorithm (GA) [12].

Furthermore, Zhou et al. presented a study in whichthe K-mean algorithm was used to label the un-labelled signals [13]. A comparison of the performance between ANN and KNN was carried out by Gunerkar et al. [14]. The study was performed on rolling element bearings based upon a feature extracted after wavelet transform. J. P. Patel compared the performance of ANN with SVM for bearing fault diagnosis [15]. Tian et al. presented a bearing defect diagnosis method based on spectral kurtosis and cross correlation [16]. The method was validated by experiments using a machinery fault simulator. R. Zhang et al. undertook a fault diagnosis of a rolling element bearing using a k-mean singular value decomposition dictionary learning algorithm and a back propagation neural network [17]. Amar et al. used vibration spectrum imaging to train the ANN classifier for bearing fault diagnosis [18]. Khan and Kim trained a KNN classifier for bearing fault diagnosis under different speeds using features extracted from grayscale vibration images. The grey scale image textures were encrypted using the local binary pattern operator [19]. Ankush Mehta et al. used a combination of infrared thermography and different machine learning techniques for bearing fault diagnosis. They used two-dimensional discrete wavelet transform for feature extraction from thermal images. PCA was used for dimensionality reduction. KNN, linear discriminant analysis, and SVM were used as classifiers. A comparison of the performance of all three classifiers was also carried out [20]. Wu et al. proposed a bearing fault diagnosis technique through kernel matrix construction-based support vector machine [21].

The process of fault diagnosis through conventional machine learning techniques mainly consists of data acquisition, features extraction, features selection and finally fault classification or health state prediction. Classical machine learning algorithms are considered superficial because of their limited learning and generalization ability; therefore, they cannot extract important information from large, complex, and non-linear data. Although classical machine learning techniques have reduced human intervention in the fault diagnosis process, to apply classical ML techniques for bearing fault diagnosis, the correct features extraction and selection process is crucial, and this is still a labor-oriented exercise and requires expert knowledge to some extent. To further reduce human intervention in the bearing fault diagnosis process, techniques based on deep learning theories have been implemented. These deep learning-based techniques include autoencoder (AE) -based techniques, deep belief network (DBN) -based techniques, and convolutional neural network-based techniques. AE- and DBN-based techniques are unsupervised techniques and are mostly used for features extraction and information fusion. Models based on these techniques can be trained on unlabeled data and for labelled data fine tuning is required. As they are unsupervised techniques, AE-based techniques cannot be used directly for bearing health state identification, and a classification layer needs to be added at the top of the model architecture [22]. Whereas CNN-based techniques are supervised techniques and they are task specific. Due to their capability of joint features and classification learning, they can achieve better classification accuracy as compared to other deep learning models in image classification problems [23]. Deep learning models can automatically extract features from vibration data and manual features extraction is not required. Thus, deep learning techniques can provide end-to-end diagnosis models to further reduce human intervention in defect diagnosis activities. Deep learning models have gained the attention of researchers for bearing defect diagnosis, such as, the deep neural network (DNN) -based scheme for rolling element bearing fault diagnosis [24], the DNN for fault characteristic mining and the intelligent diagnosis of rotating machinery with massive data [25], the short-time Fourier transform-deep learning scheme for rolling bearing fault diagnosis [26], the bearing condition recognition method based on multi-feature extraction and DNN for the intelligent condition monitoring of bearings [27], the multi vibration signals and DBN scheme for bearing fault diagnosis [28], the CNN-based method for bearing and rotor fault detection [29], the hierarchical adaptive deep CNN approach for bearing fault diagnosis [30], the energy-fluctuated multiscale feature mining technique based on a wavelet packet energy image and a deep convolutional network for spindle bearing fault diagnosis [31], and the deep residual learning-based method for bearing fault diagnosis [32]. Moreover, Saucedo-Dorantes et al. [33] presented a fault diagnosis methodology for different bearing technologies (metallic, hybrid and ceramic bearings) based on deep feature learning. Vibration and current data were acquired under different operating conditions. Time domain, Frequency domain and Time Frequency domain features were extracted from vibration and current signals. A staked auto encoder-based feature learning method was introduced. Information from different domains was integrated through feature fusion and softmax layer was used for final classification. Ma et al. [34] presented a multi source information fusion algorithm based on variational autoencoder and random forest for bearing fault diagnosis in case of limited labeling. Hoang and Kang [35] proposed a bearing fault diagnosis method based upon motor current signal, using CNN and the information fusion technique. CNN is used for automatic features extraction from grey images from the current signal. After the application of the information fusion technique, classical machine learning algorithms are used for classification. Three working conditions are considered in this study. However, training and testing is performed under the same operating conditions.

Under the umbrella of deep learning, convolutional neural networks are a special type of DNNs which are known for their image recognition and classification capability. Researchers have used one dimensional (1D) and two dimensional (2D) CNN models for bearing defect diagnosis. In the 1D-CNN model, raw vibration data can be directly used as input to the model, as used by Eren et al. [36,37]. For 2D-CNN models, vibration data cannot be used in their raw form. Rather, they are initially converted into time-frequency image representations such as spectrograms [38,39,40], scalograms [40,41] or other types of vibration images [42]. Then these images which represent the vibration signal in image form are used as input in 2D-CNN model.

For conventional ML models and deep learning models, generally it is assumed that training and testing data sets belong to the same operating conditions. The performance of these models may degrade significantly if the operating conditions under which the models are deployed for prediction differ from the operating conditions under which the models were trained [43]. In real world applications, such as in trains, bearings may undergo different speed and loading conditions. Due to varying speed, the frequency characteristics of vibration signal also change. Therefore, accurate intelligent defect diagnosis of rolling element bearings under variable/inconsistent operating conditions is still a challenge for researchers. Moreover, enough training data may not be available for all potential operating conditions. This situation arises frequently which makes it challenging to use AI methods for fault diagnosis outside the laboratory environment. To solve this issue, transfer learning was introduced by researchers [44]. Zhang et al. proposed a transfer learning approach with neural networks for bearing fault diagnosis in changing working conditions when there is only a small amount of target data available [45]. Shen et al. presented a singular value decomposition-based features extraction and transfer learning-based approach for bearing fault diagnosis under various operating conditions [46]. Chao et al. presented an enhanced least squares support vector machine-based transfer learning approach for bearing fault diagnosis in the case of small target dataset availability [47]. Chunfeng et al. proposed a heterogeneous transfer learning-based approach for the scenarios when the available labelled data in the target domain is less [48]. Cheng et al. presented a deep adversarial transfer learning method based on Wasserstein distance for bearing fault diagnosis with insufficient labelled data [49]. However, most of the studies in transfer learning have normally considered some fixed operating conditions. Whereas, practically, the operating condition may lie between these fixed conditions. Furthermore, in most of the studies there is still a need for a small amount of labelled target domain data.

For bearing fault identification under variable speed, Bruand et al. reconstructed shaft orbits using angle measurements and derived a feature based on shaft orbit shape. It was shown that the signature of the rolling element bearing fault can be observed in shaft orbit. The information was retrieved using a set of angle measurement sensors. In order to remove speed variation, angular reference was used to work in the angular domain [50]. Order tracking-based methods have gained the attention of researchers for rolling element bearing diagnosis under variable conditions. Mishra et al. proposed a technique to use order tracking on the envelop of wavelet denoised estimate of the short duration angle synchronous averaged signal for bearing fault diagnosis under variable conditions. To extract deterministic content from the vibration signal, the Bayesian wavelet denoising approach was adapted and envelop order spectra were utilized to identify the faults [51]. Guo et al. proposed a rolling element bearing fault detection method under variable speed based upon envelop order tracking utilizing envelop analysis, order tracking and spectral kurtosis [52]. However, these methods cannot be used for automatic defect diagnosis as they are not intelligent methods and require human expertise in the field. For the automatic fault diagnosis of roller bearings under variable speed, Yang et al. extracted the features from a vibration signal by combining local mean decomposition and order tracking techniques. The features’ values were used as the input to a variable predictive model-based class discriminate classifier for automatic fault pattern and working condition identification [53]. Farhat et al. updated three frequency domain features, spectrum peak ratio outer, spectrum peak ratio inner, and spectrum peak ratio rolling element, to perform with a nonstationary signal, utilizing the order tracking technique. The updated features were used as an input to a multi-kernel support vector machine classifier for automatic defect classification [54]. However, these order tracking-based methods involve manual features extraction and a modification process.

To avoid the manual features extraction process, researchers have proposed end to end bearing diagnosis techniques based on CNN. Appana et al. proposed a low-speed bearing fault diagnosis method using CNN and envelope spectrums extracted from acoustic emission signal. The maximum speed considered was 500 revolutions per minute (rpm) [55]. Pham et al. presented a fault classification method utilizing the spectrogram images and CNN under four different speeds 1730 rpm, 1750 rpm, 1772 rpm and 1797 rpm [39]. In another study Pham et al. presented a fault classification method under different rotational speeds between 250 rpm and 500 rpm. Spectrogram images and CNN were utilized [38].

Researchers have mainly undertaken the defect diagnosis of bearings under variable operating conditions considering only one operating condition i.e., speed. However, bearings may experience variable loading conditions in some applications. Furthermore, speed variation is too low, for example between 1730 and 1797 rpm, or the proposed model is tested only on low speeds. Moreover, the considered speeds are steady; ramp or varying conditions are not considered. However, in real world applications, such as trains, continuously varyingied speed may be experienced which includes acceleration and deceleration. Furthermore, seeded defects are mostly researched and these may be an easier task for the machine learning or deep learning model as compared to diagnosesing the bearing defects which are encountered during real field operation. Therefore, there is a need to propose a robust method for bearing defect diagnosis under variable operating conditions, which can perform the task of fluctuations of speed and load. Moreover, to access the training data from all potential operating conditions may not be possible for most real-world applications. Therefore, it is necessary to find some signal properties which are invariant to changing operating conditions and only change due to changing health state so that AI models trained under one set of operating conditions may correctly predict, under different operating conditions, whose fault data for training is not available.

In this research a novel method for the fault diagnosis of rolling element bearings is proposed utilizing a combination of the order analysis and image classification and recognition capabilities of CNN. Order maps are computed from the vibration signal of rolling element bearings. These order maps show consistent properties over varying operating conditions. They vary with respect to the type of defect but show consistent configurations under changing operating conditions, therefore, they can be considered as operating condition (speed and load) invariant vibration images. The order maps computed under one set of operating condition are used to train the CNN model. The trained CNN model is deployed for defect diagnosis under different operating conditions (speed and load). Performance of the proposed method is compared with five techniques: (1) a technique based on spectrograms and CNN as utilized in [38,39,40], (2) KNN in combination with GA as utilized by [12], (3) ANN/multilayer perceptron, (4) SVM, (and5) KNN + +order maps.

## 2. Proposed Defect Diagnosis Methodology

The intelligent fault diagnosis of rolling element bearings using a vibration signal requires features extraction if we use conventional machine learning techniques. For accurate diagnosis, correct features extraction and selection is of paramount importance [12]. However, deep learning models such as convolutional neural networks (2D-CNN) have the capability to learn discriminative features directly from vibration characteristic images such as spectrograms, scalograms etc. Therefore, tedious features extraction and selection can be avoided by using CNN models for fault diagnosis. However, due to a change in operating conditions, the vibration images of the signal may also change, resulting in degradation of the performance of the CNN model for fault diagnosis under different operating conditions. Therefore, in this study a fault diagnosis method for rolling element bearings is proposed based upon the operating conditions of invariant vibration images and a deep convolutional neural network. Variation in two operating conditions: speed and load, are considered in this study.

Vibration signals of locomotive rolling element bearings are acquired under different speeds (steady and variable) and loads. Large fluctuations of speed including transient conditions are considered. Tachometer signal is also simultaneously acquired. Different types of real field and seeded defects of locomotive rolling element bearings, including combined defects, are considered in this study. Vibration signals are segmented into 2 segments with an overlap of 1.6 s. Order maps are computed from each segment. These order maps are used as input for CNN model which automatically learns the features from these images/order maps to classify the different defect types of rolling element bearings.

**Figure 1:**
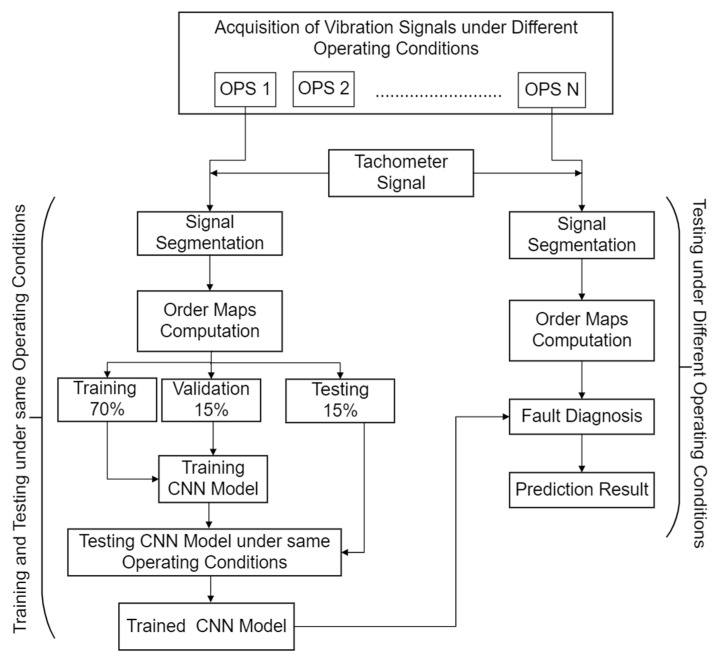
Flow chart of proposed methodology.

Initially, the CNN model is trained and tested under the same operating conditions. Data are divided into the training, validation, and testing data sets in the ratio of 70%, 15% and 15%, respectively. After completing the training and testing process under the same operating conditions, the trained model is deployed for defect diagnosis under all of the other operating conditions considered in this study. The same process is repeated for the other combinations of speed and load as well. The proposed methodology is depicted in Figure 1. Details of order maps computation, the CNN model and its implementation are given in following subsections:

### 2.1. Order Maps

For rolling element bearings’ fault diagnosis, characteristic frequencies play an important role for identifying a specific type of defect. As frequency indicates the repetition times per second, in the case of varying shaft speed, spectral line smearing may cause difficulty in fault diagnosis using spectrograms [56]. Consequently, traditional Fourier Transform-based methods are no longer effective for bearing fault diagnosis under varying rotational speed. Thus, the time-frequency vibration images generated from the vibration signal, based on short-time Fourier Transform such as spectrograms will also undergo change if the shaft speed is changed. Consequently, a CNN model trained on such vibration images for fault diagnosis may not be able to predict accurately under varying speed.

**Figure 2:**
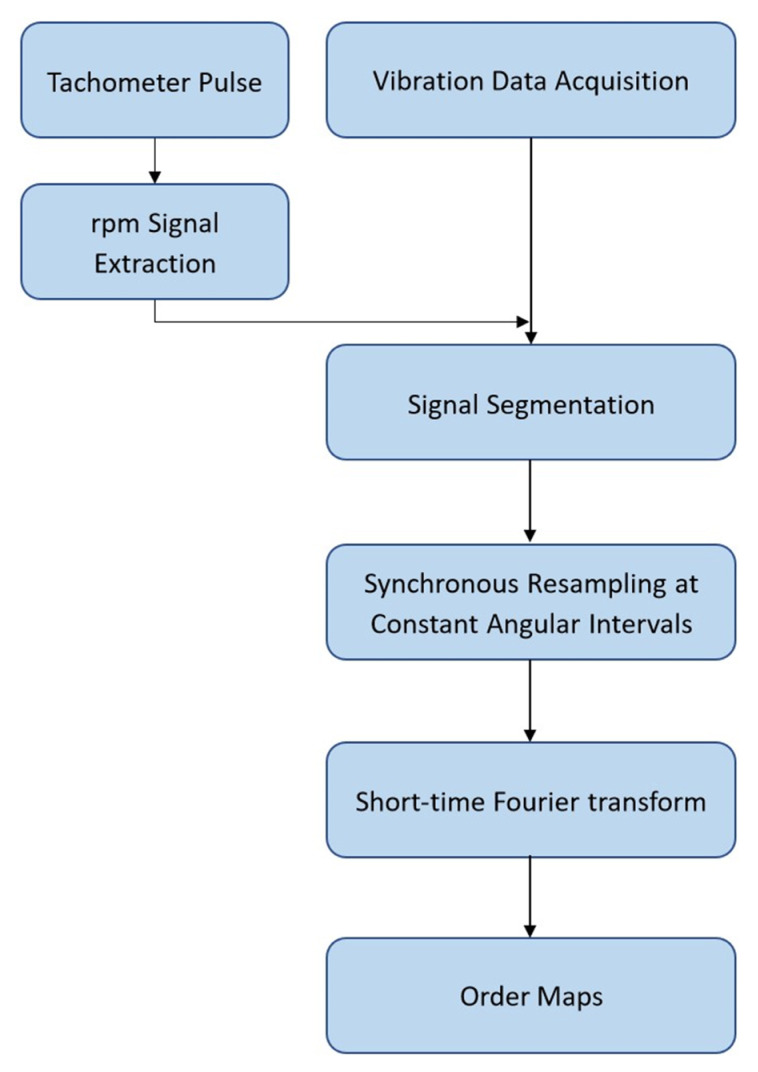
Order maps’ computation procedure.

The problem can be solved by converting a non-stationary signal into a stationary signal in the angular domain by resampling at constant angular intervals. This technique is called order tracking. An order indicates a frequency which is a specific multiple of the rotational speed. Order tracking has been identified as a trustworthy and practical approach to mitigate the effects of spectral line smearing caused by varying rotational speed [57,58]. In this study, order maps are computed from the vibration signals utilizing the tachometer pulse according to the procedure shown in Figure 2. These order maps show consistent patterns under varying speed and are used as input to the CNN model.

Order maps computation mainly involves the following three sections:

#### 2.1.1. Tachometer Signal Processing and rpm Extraction

Tachometer signal processing is very important in order tracking. If a good stable tachometer signal is not available, then accurate order maps cannot be computed [59]. Tachometer pulse is converted into an rpm signal as given by Equation (1) [60].

(1) $$rpm\left(t\right)=\frac{60}{N_{p}\left(t2-t1\right)}$$

where, $N_{p}$ is number of pulses per revolution and (t2−t1) is the time instance between two pulses. For this purpose, initially low and high states of tachometer signal are determined. Time for each pulse is determined by averaging the start and end time readings of pulse. Time interval between pulse centres is determined to find the rpm at the interval midpoint by *rpm* = 60/Δt. Afterwards, instantaneous rpm values are interpolated linearly onto the time axis of original signal.

#### 2.1.2. Synchronous Resampling in the Order Domain

Phase angle (A(t)) is determined as the time integral of rotational speed as shown in Equation (2).

(2) $$A\left(t\right)=\int_{0}^{t}\frac{rpm\left(t\right)}{60}dt$$

The vibration signal is resampled onto the new time axis instead of the original constant time axis. Resampling of the vibration signal is done at non sampled time points by utilizing an upsampled vibration signal and interpolating it linearly. Mostly, 10–20 times oversampling gives adequately good accuracy [60]. In this study signal is upsampled by a factor of 15 and then linearly interpolated onto a consistent grid in the phase domain. Thus, a constant number of samples per cycle is achieved to produce a stationary sinusoid for each order. After synchronous resampling, the vibration signal comes in the order domain. The signal frequencies which are constant multiples of the rotational speed are now converted into constant tones. The smearing of spectral components which occurs due to rapid frequency change with time, reduces because of this transformation. The relationship between the highest accessible order ($O_{max}$), sampling frequency ($f_{s}$) and the maximum rotational speed of the shaft is given by Equation (3).

(3) $$O_{max}=\frac{f_{s}}{2\left(\frac{\max\left(rpm\right)}{60}\right)}$$

To accurately capture the maximum order, the angular domain sampling rate $\left(f_{a}\right)$ must be at least two times that of $O_{max}$. i.e., $f_{a}\geq 2*O_{max}$.

#### 2.1.3. Short-Time Fourier Transform of Resampled Signal in the Order Domain

**Figure 3:**
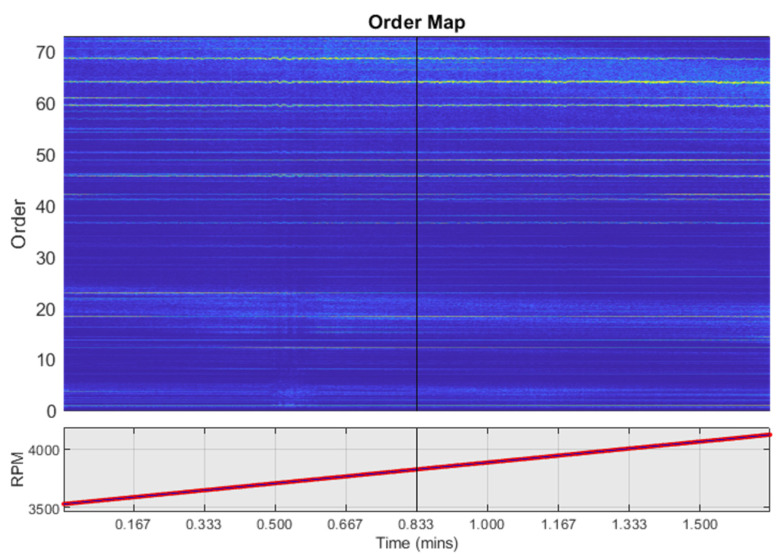
Order map under variable speed (3525–4125 rpm).

Short-time Fourier transform of the interpolated signal is computed to generate a spectral map of order versus rpm. Since each order is a fixed multiple of the reference rotational speed, the order map has a straight track as a function of rpm for each order, as shown in Figure 3. Therefore, these maps show consistent patterns under varying speed. The maximum order which can be accurately captured is dependent on sampling frequency and maximum rotational speed, as shown by Equation (3). Therefore, to use the order map images as input to a CNN, it is essential to keep the same maximum order limit for the whole diagnosis process lower than the $O_{max}$ related to maximum speed under consideration and the sampling frequency.

**Figure 4:**
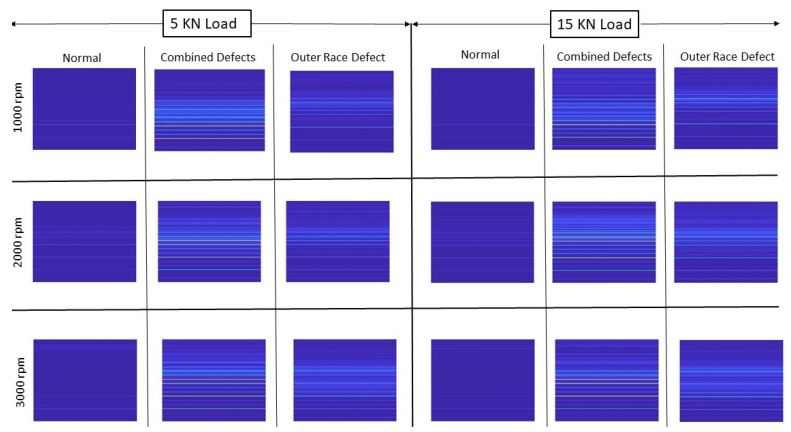
Order maps of bearings with different types of defects at different speeds and loads.

To ensure the normal operation of rolling element bearings, sufficient friction force is required by the race ways. If the bearing is not properly loaded, slip page may appear in the contact zone and relative skid may occur between the rolling elements and the inner/outer race ways [61]. This phenomenon generally occurs in low loaded roller bearings [62]. Because of this phenomenon, frequencies may be slightly affected [63], which in return may affect the order maps. Consequently, performance of the deep CNN model for fault diagnosis under different loading conditions may degrade. Han et al. studied the skidding behavior of cylindrical roller bearings under variable loads. It was reported that an increase in radial load and bending moment reduces the skidding. By increasing the load, the friction force of the rollers increases, and the maximum slipping velocity of the rollers reduces, leading to the pure rolling rotation of the cage. Therefore, after certain radial load, no overall skidding phenomenon was present in the bearing [64]. Feng et al. reported that an increase in radial load can reduce the skidding of ball bearings [65]. Deep groove ball bearings are less sensitive to skidding because of an extra degree of freedom of the rolling elements. Therefore, if the bearing is properly loaded and diagnosis is not performed under a load or very low loading conditions, the order maps may not be significantly affected, and the CNN model will perform defect diagnosis correctly under the changing loading conditions as well. The effect of load change on the order maps for deep groove ball bearings is observed experimentally. Order maps of a healthy bearing, a bearing with combined defects and a bearing with outer race defect are shown in Figure 4 for three different speeds (1000 rpm, 2000 rpm and 3000 rpm) and two different loads (5 KN and 15 KN) which are the minimum and maximum loads considered in this study. It can be observed that the patterns of order maps do not change with changing speed and load. However, they show different patterns for different fault conditions, making themselves insensitive to operating conditions (speed and load) but fault discriminative. Therefore, they will be used for fault diagnosis under variable speeds and loads.

### 2.2. Convolutional Neural Network

Convolutional neural networks are known for their high capability in the field of image identification and classification. They consist of an input layer, hidden layers, and an output layer. Generally hidden layers in convolutional neural networks consist of convolutional layers, Rectified Linear Linear Unit (ReLU) layers, pooling or subsampling layers and fully connected layers [22]. The 2D-convolutional layer uses its kernels to convolve the input by moving the kernels vertically and horizontally and getting the dot product of kernels and input and then addings a bias term. Its input is the output of the previous layer. Kernels extract the local features of the input region. An activation function, such as ReLU, is used to obtain output from the results of the convolutional operation which are called features. Sometimes ReLU is shown as a separate layer from the convolutional layer. The ReLU layer performs a threshold operation in which all the values less than zero are set to zero. The mathematical model of the convolutional layer is given in Equation (4).

(4) $$X_{j}^{m}=f\left(\sum_{i\in C_{j}}X_{i}^{m-1}*k_{ij}^{m}+b_{j}^{m}\right)$$

$C_{j}$ represents the input map selection, *m* is the *m*th layer in the network. $X_{i}^{m-1}$ is the input of the convolutional channel. k is the kernel matrix, and b is the bias matrix. f is a nonlinear activation function such as ReLU. After the convolutional layer, a pooling or subsampling layer is applied which reduces the size of the input features and network parameters. The pooling layer can be represented by Equation (5).

(5) $$X_{j}^{m}=f\left(\beta_{j}^{m}\;down\left(X_{j}^{m-1}+b_{j}^{m}\right)\right)$$

where, down (.) represents the pooling function. $b_{j}^{m}$ is additive bias and $\beta_{j}^{m}$ is multiplicative bias. Based upon the pooling function, the pooling operation can be maximum pooling or average pooling. After staking the convolutional and pooling layers multiple times, the output is fed to a fully connected layer at the final stage. It is a feed forward neural network (multi-layer perceptron) which uses softmax as an activation function in the output. The softmax activation function can be described by Equation (6).

(6) $$\sigma {\left(\vec{y}\right)}_{i}=\frac{e^{y_{i}}}{\sum_{j=1}^{N}e^{y_{j}}}$$

where, σ is the softmax, $\vec{y}$ is the input, $e^{y_{i}}$ is the exponential function for the input, $e^{y_{j}}$ is the exponential function for the output and N is the number of classes. The purpose of the fully connected layer is to collect all the features learned from the previous layers to identify patterns. Therefore, all the neurons in the fully connected layer are connected to all neurons in the previous layer. Moreover, in order to increase the training speed of the convolutional neural network and to reduce the sensitivity to network initialization, a batch normalization layer is used between the convolutional layer and nonlinearities. This layer independently normalizes the minibatch data across all channels. Furthermore, to avoid the possibility of the network memorizeing some specific features, a dropout layer can be added prior to the fully connected layer which sets the input to zero as per set probability.

**Figure 5:**
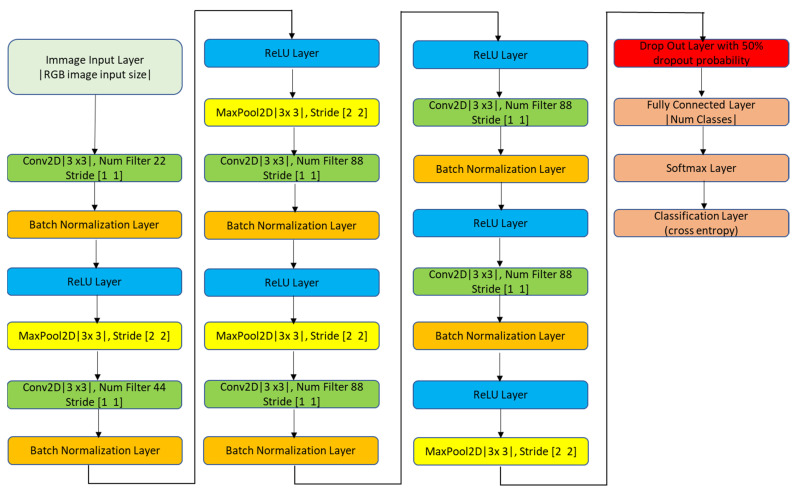
Architecture of proposed convolutional neural network.

The convolutional neural network architecture proposed in this study is shown in Figure 5. The order maps were saved in Bitmap Image file format in original size (656 × 875 × 3) without compression to avoid loss of information. Batch normalization layers are added between 2D-convolutional layers and ReLU layers. A combination of the 2D convolutional layer, the batch normalization layer and the ReLU layer makes one convolutional block. Overlapping max-pooling layers are added after 1st, 2nd, 3rd, the first second third and sixth convolutional blocks. A dropout layer with 50% drop out probability is added before the fully connected layer in order to avoid over fitting. Adaptive moment estimation optimizer is used for optimization of hyperparameters because of lesser memory and tuning requirement and faster optimization capability as compared to other optimization algorithms [40]. The initial learn rate was set as 0.0003 and it was dropped by a factor of 0.1 after every 10 epochs. The minibatch size was set as 15.

## 3. Experimental Setup

The proposed method for defect diagnosis of rolling element bearings under different operating conditions was validated using the vibration data from locomotive rolling element bearings. A total of two case studies were conducted utilizing two different test rigs developed to test bearings for railway electrical traction. Details of the same are appended below.

### 3.1. Case Study-1

**Figure 6:**
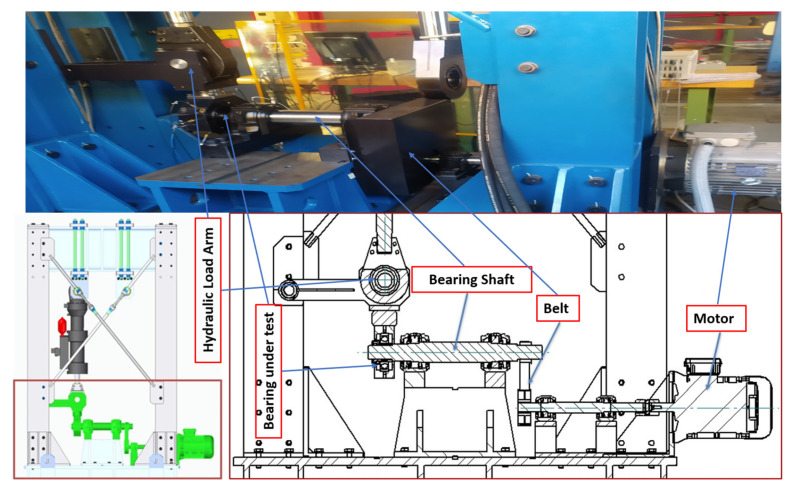
Test rig-1 layout.

**Figure 7:**
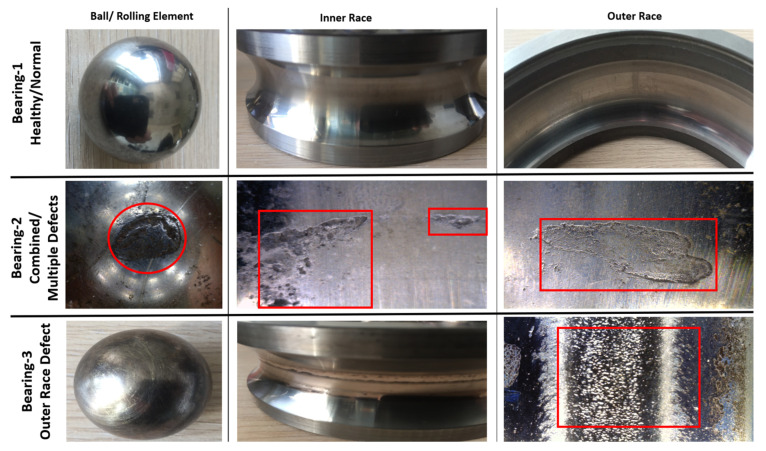
Healthy and defected bearings considered in case study-1.

**Figure 8:**
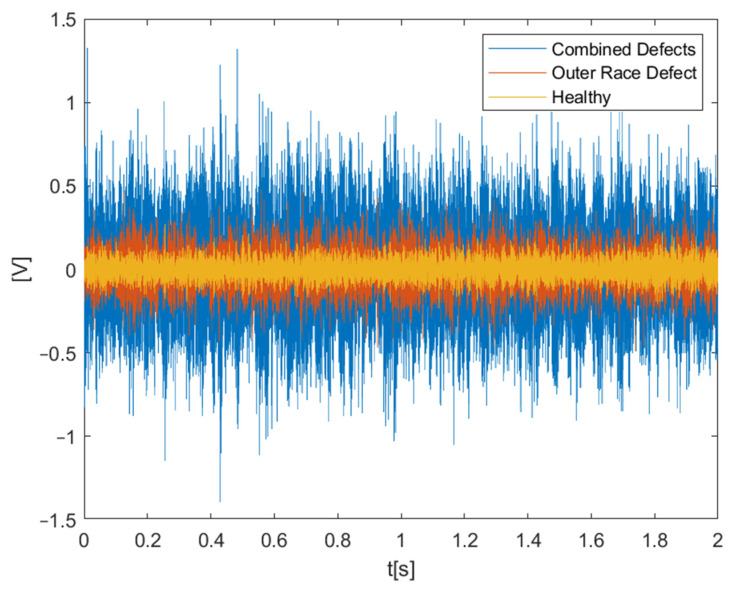
Raw vibration signal in the time domain.

The first test rig (shown in Figure 6) was designed to test locomotive motor bearings under different operating conditions (speed and load). Operating speed and load can be varied in order to get data under different operating conditions. Vibration data were acquired at 25,600 Hz sampling frequency by two accelerometers installed at different angular positions in radial direction. A tachometer was installed at the bearing shaft to give one pulse per revolution signal. For each operating condition, data were acquired for 50 s. SKF 6318, deep groove ball bearings were tested under nine different operating conditions which are described in Table 1. A total of three health states including one normal and two faulty conditions with real field defects are considered in this study, as shown in Figure 7 and Table 2. Raw vibration signals for all three types of bearings considered in case study-1, at 1000 rpm and 5 KN load, are shown in Figure 8.

### 3.2. Case Study-2

**Figure 9:**
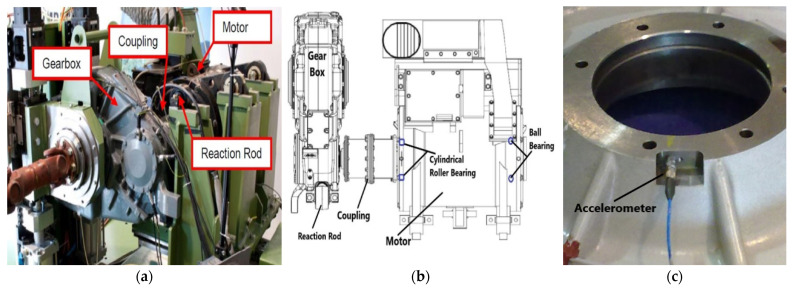
Test rig-2 layout. (**a**) main components; (**b**) under study bearings’ position; (**c**) accelerometer’s position.

**Figure 10:**
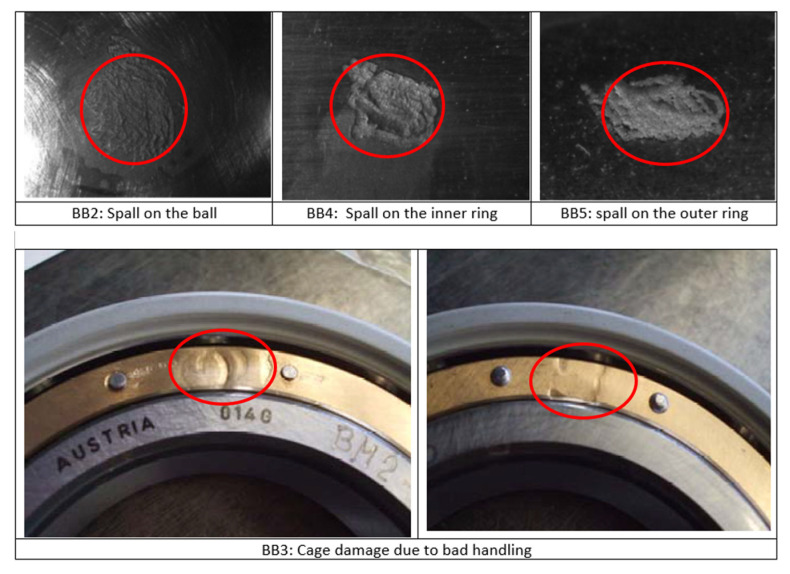
Different types of seeded defects of ball bearings (case study-2).

**Figure 11:**
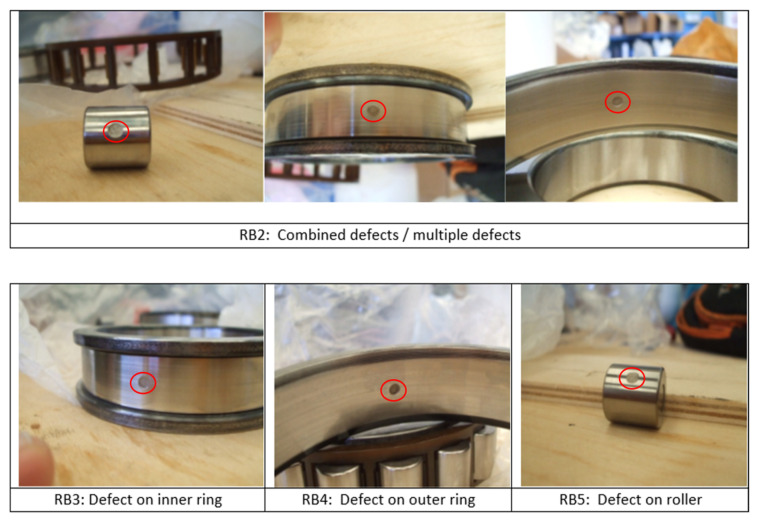
Different types of seeded defects of cylindrical roller bearings (case study-2).

An actual traction system of a high-speed train with an ability to reach 250 km/h speed was installed on the test bench, as shown in Figure 9 [66]. The traction motor is 265 kW converter driven 4-poles asynchronous motor. The rotor of the traction motor is mounted on two types of bearings: a single row, groove ball bearing (BB), SKF-6214, and a cylindrical roller bearing (RB), SKF-N214, on the driven end, as shown in Figure 9. Vibration data at 20,000 Hz sampling frequency are acquired for both support bearings of the traction motor at different speeds (constant speed, acceleration, and deceleration), as presented in Table 3. Accelerometers were installed in the proximity of the bearings, on the inner surface of motor flanges holding the respective bearings, as shown in Figure 9. Tachometer signal was also acquired simultaneously. For both types of the bearings, one healthy and four defective bearings with seeded defects of different types were considered in this study, as shown in Figure 10 and Figure 11 and Table 4.

## 4. Results and Discussion

**Figure 12:**
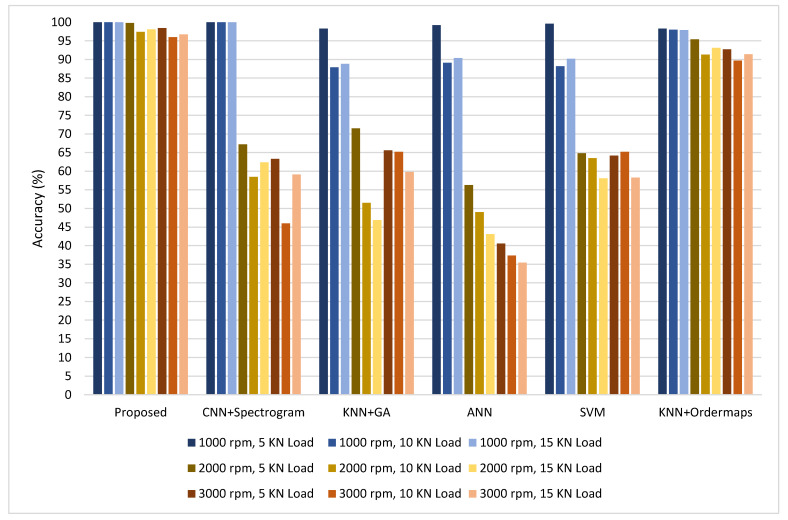
Performance comparison when models were trained at 1000 rpm + 5 KN load and tested under all combinations of speed and load.

During the first case study on test rig 1, vibration data were acquired using two accelerometers under nine different operating conditions (different combinations of speed and load). One healthy and two defective deep groove ball bearings with outer race defects and combined defects were considered. Vibration signals were divided into segments of 2 s with an overlap of 1.6 s. Order maps were computed from the vibration signals of both sensors separately and were combined to train the proposed CNN model for fault diagnosis under one set of operating conditions. The trained model was tested for defect diagnosis under all of the other combinations of speed and load considered during the first case study. The same procedure was repeated for all sets of operating conditions considered in this study. The performance of the proposed methodology for defect diagnosis under different operating conditions in terms of prediction accuracy is given in Table 5, and the same is compared with five methods: (1) the methodology used in [38,39,40], in which time frequency images and spectrograms were used as input to the CNN, (2) KNN as a classifier in combination with GA for features selection using the time domain and spectral kurtosis-based features as utilized in [12], (3) ANN/multilayer perceptron using the same features as utilized in [12], (4) SVM using the same features as utilized in [12], and (5) KNN + order maps using Histogram of Oriented Gradient (HOG) features. Initially, the models were trained at 1000 rpm and 5 KN load. The trained models were deployed for predictions under all other combinations of speed and load. The proposed CNN model predicted with 100% accuracy under all loading conditions at 1000 rpm for both cases i.e., with order maps and spectrograms. Afterwards, for spectrograms, performance degraded drastically when the model trained at 1000 rpm and 5 KN load was deployed for predictions at 2000 rpm (5, 10 and 15 KN load) and 3000 (5, 10 and 15 KN load). However, performance of the proposed methodology in terms of fault diagnosis/prediction accuracy remained excellent under all the loading conditions at 2000 rpm and 3000 rpm with a minimum fault diagnosis accuracy of 96% at 3000 rpm and 10 KN load. However, the minimum fault diagnosis accuracy was 46% when spectrograms were used as input for the CNN model. The fault diagnosis accuracy ofthe other four methods considered for comparison i.e., KNN + GA, ANN, SVM and KNN + order maps was 98.3%, 99.2%, 99.6% and 98.3%, respectively, under thesame operating conditions (when the models were trained and tested at 1000 rpm and 5 KN load). However, when these trained models were deployed for diagnosis under different operating conditions as compared to those under which they were trained, their performance degraded drastically except for KNN + order maps. By changing the load under same speed, the performance of KNN + GA, ANN and SVM was degraded to 87.9%, 89.1% and 88.2%, respectively. However, performance degradation was more drastic by changing the speed which dropped to 46.9%, 35.4% and 58.3% for KNN, ANN and SVM, respectively. For KNN + order maps the performance degradation was not significant by changing the load, however, fault diagnosis accuracy dropped to a minimum of 89.7% by changing the speed which is much better than KNN + GA, ANN, SVM and CNN + spectrograms. However, the proposed method performed better as compared to KNN + order maps. A performance comparison of all models when trained at 1000 rpm and 5 KN load is given in Figure 12.

The same trend was observed when the procedure of training under one set of operating conditions and testing under all other combinations of operating conditions was repeated for all operating conditions considered in this study. It is observed that change in the loading condition at steady speed did not affect the performance of the CNN model in both cases i.e., with order maps and with spectrograms. Similarly, the performance of the KNN model with order maps was also not affected greatly by changing the load. This depicts that spectrograms and order maps are not affected much by changing the load. Although, the performance of KNN + GA, ANN and SVM models was degraded at an average of about 13%. When the speed under which the models were trained differed from the speed under which the models were deployed to make predictions, the performance of the CNN model was adversely affected in the case of spectrograms, which shows that by changing the operating speed spectrograms undergo change and thus the performance of the CNN model for fault diagnosis is affected. Similarly, drastic performance degradation was observed for KNN + GA, ANN and SVM models. The performance of KNN + order maps also degraded but not drastically. However, the proposed methodology showed very good performance under changing speed as well, because of the invariant behaviour of order maps under varying speeds the good image classification and recognition ability of the deep CNN model. The proposed method did diagnosis the faults of a locomotive rolling element bearing under different operating conditions with an overall average accuracy of 98.4% which is much higher compared to the average accuracy of 73.7%, 67.4%, 64.7% and 65.3% for CNN + spectrograms, KNN + GA, ANN and SVM, respectively. For KNN + order maps the overall average prediction accuracy was 92.3% which is much higher compared to other methods used for comparison, but it is less than the average fault detection accuracy of the proposed methodology. The explanation of the better performance of CNN and KNN in the case of order maps is that the order maps exhibit consistent patterns under variable speed, as described in Section 2.1, and for deep groove ball bearings under the load range considered in this study they did not exhibit much change. Therefore, the CNN and KNN models were able to correctly classify them. However, because of the better image classification abilities of the proposed deep CNN model, the proposed method performed better as compared to KNN + order maps. In order to use the KNN model for this fault diagnosis task we had to extract HOG features from order maps, whereas the deep CNN model can automatically learn discriminative features from the order maps for defect diagnosis under variable speeds and loads with high accuracy.

**Figure 13:**
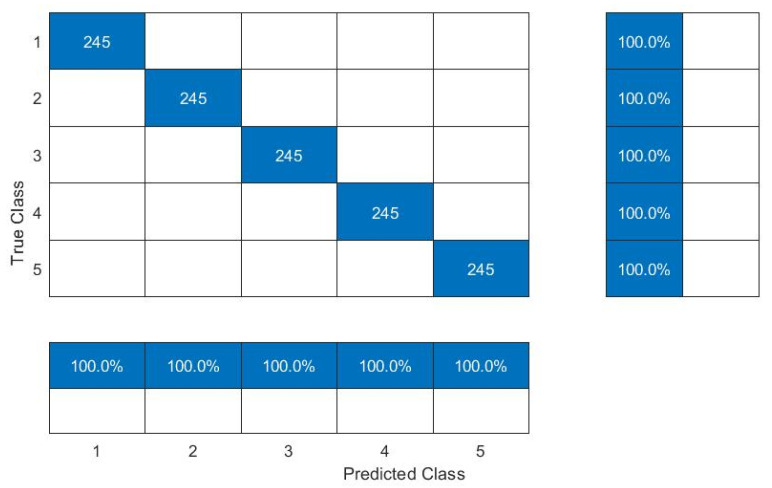
Confusion matrix: model trained at 3170 rpm and tested at 4955 rpm.

**Figure 14:**
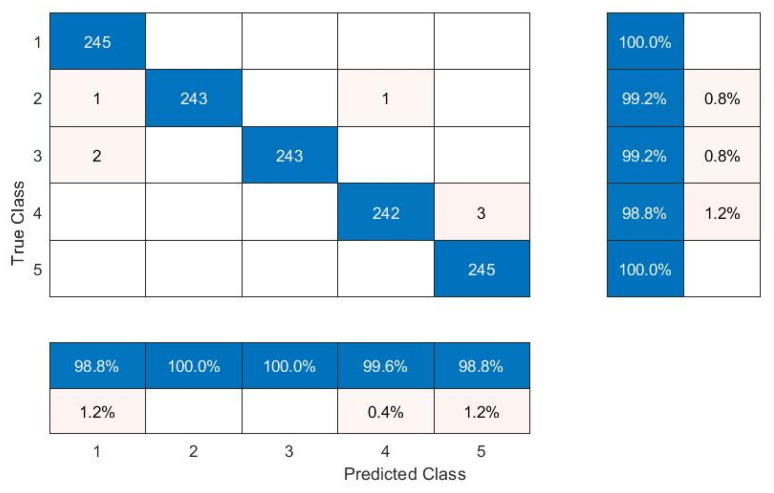
Confusion matrix: model trained at variable speed between 1000 and 2000 rpm and tested at variable speed between 3525 and 4125 rpm.

In the second case study, vibration data were acquired for two types of rolling element bearings installed in the locomotive traction motor, under five different variable speed conditions (constant speed, acceleration, and deceleration). The load was constant which corresponds to the motor shaft weight. Order maps were computed from vibration signal segments of 2 s to use as input for the proposed CNN model. An overlap of 1.6 sec was used for each segment. Initially, the CNN model was trained at a steady speed of 3170 rpm and was tested under all other operating conditions considered during this study. Later, the model was trained under the transient condition of variable speed between 1000 and 2000 rpm, and was deployed for defect diagnosis under all other operating conditions considered in this study. The performance of the proposed methodology in terms of prediction accuracy is given in Table 6. The proposed defect diagnosis method undertook a fault diagnosis of ball bearings and cylindrical roller element bearings installed in the locomotive traction motor under variable speeds with an average accuracy of 99.2% and 98%, respectively. In this case study, the overall accuracy of the proposed methodology for defect diagnosis of rolling element bearings under variable speed remained 98.6%, which proves that the proposed method is capable for the defect diagnosis of rolling element bearings with good accuracy at a high speed with large variations. A confusion matrix for ball bearings, when the model was trained at 3170 rpm and tested at 4955 rpm is shown in Figure 13, and when the model was trained at a variable speed between 1000 and 2000 rpm and tested at variable speed between 3525 and 4125 rpm is shown in Figure 14.

For the implementation of the proposed methodology, in addition to the vibration signal, a tachometer signal is also required. However, most of the other methods, including thefour methods used for comparison in first case study i.e., CNN + spectrograms, KNN + GA, ANN and SVM, do not require a tachometer signal. Therefore, the necessity of a very accurate tachometer signal is the limitation of the proposed methodology. If there is an error in the tachometer, then resampling may fail and correct order maps cannot be computed, which will adversely affect the diagnosis performance of the proposed methodology.

## 5. Conclusions

In this study a method for the defect diagnosis of rolling element bearings under variable operating conditions (speed and load) using order maps and convolutional neural networks is proposed. A non stationery signal was resampled synchronously at con-stant angular intervals to convert it into a stationery signal in the order domain. Short-time Fourier Transform of the resampled signal was computed to generate the order maps. These maps show consistent patterns under variable speed but different patterns for different types of defects. The sensitivity of order maps for changing load was studied experimentally for deep groove ball bearings and it was found that they remain consistent under varying loads if the bearings are properly loaded. Therefore, order maps can be termed as operating condition (speed and load) invariant but fault discriminative vibration images. In addition, due to this property, they can be used for fault diagnosis under varying speeds and loads. A deep CNN model was proposed which can automatically extract fault discriminating features from the order maps for defect classification. Order maps were used as input to the CNN model for fault diagnosis under varying speeds and loads. The proposed method conducted the fault diagnosis of different types of locomotive rolling element bearings under a huge fluctuation of operating conditions (speed and load) with an average prediction accuracy of 98.4% and 98.6% in two separate case studies. The proposed method outperformed when it was compared with other CNN, KNN, ANN and SVM-based methods. The limitation of the proposed method is the requirement of a very accurate tachometer signal. The proposed method can be implemented for rolling element bearings’ fault diagnosis under variable speeds and loads, such as in the transportation industry. In future studies, the efficacy of the proposed methodology needs to be investigated for such applications where a tachometer signal is not available along with the vibration signal, by extracting the rpm information from the vibration signal using signal processing techniques.

## Figures and Tables

**Table 1:** Different operating conditions considered in case study-1.

Operating Condition Number	Operating Conditions		Operating Condition Number	Operating Conditions	
	Speed (rpm)	Load (KN)		Speed (rpm)	Load (KN)
1	1000	5	6	2000	15
2	1000	10	7	3000	5
3	1000	15	8	3000	10
4	2000	5	9	3000	15
5	2000	10			

**Table 2:** Bearing defect classes for case study-1.

Class	1	2	3
Health State/Type of Fault	Normal/Healthy	Combined Defects/Multiple Defects	Outer Race Defect

**Table 3:** Different operating conditions considered in case study-2.

Operating Condition Number	Type of Operating Condition	Speed Range (rpm)	Time Duration
1	Constant speed	3170	100 s
2	Constant speed	4955	100 s
3	Variable speed (acceleration)	1000–2000	100 s
4	Variable speed (acceleration)	3525–4125	100 s
5	Variable speed (deceleration)	4050–2560	100 s

**Table 4:** Bearings defect classes considered in case study-2.

Class	Defect Type/Condition	
	Ball Bearing (BB)	Roller Bearing (RB)
1	Normal/Healthy	Normal/Healthy
2	Ball defect	Combined defects
3	Cage defect	Inner race defect
4	Inner race defect	Outer race defect
5	Outer race defect	Roller defect

**Table 5:** Performance and comparison of the proposed methodology in terms of prediction accuracy for defect diagnosis under different operating conditions (case study-1).

Training		Testing		Prediction Accuracy (%)					
Speed (rpm)	Load (KN)	Speed (rpm)	Load (KN)	CNN + Order Maps (Proposed)	CNN + Spectrograms [38,39,40]	KNN + GA [12]	ANN	SVM	KNN + Order Maps
1000	5	1000	5	100	100	98.3	99.2	99.6	98.3
		1000	10	100	100	87.9	89.1	88.2	98
		1000	15	100	100	88.8	90.4	90.2	97.9
		2000	5	99.8	67.2	71.5	56.3	64.8	95.4
		2000	10	97.4	58.5	51.5	49	63.5	91.3
		2000	15	98.1	62.4	46.9	43.1	58.1	93.1
		3000	5	98.4	63.3	65.6	40.6	64.2	92.7
		3000	10	96	46	65.2	37.3	65.2	89.7
		3000	15	96.7	59.1	59.8	35.4	58.3	91.4
1000	10	1000	5	100	100	81	80.1	80.2	97.5
		1000	10	100	100	98.8	94	99.8	98.8
		1000	15	100	100	84	80.8	82.5	98.2
		2000	5	97	58	62.1	67.7	56.3	92.5
		2000	10	98.1	75.7	66.7	66.7	65.2	93
		2000	15	97.4	48.6	58.1	62.5	43.1	90.4
		3000	5	95.3	64.6	60.2	68.6	66.5	87.3
		3000	10	96.2	72.1	61.1	67.1	56.5	90.7
		3000	15	96.5	57.3	52.3	65.6	42.7	88.5
1000	15	1000	5	100	100	90.21	89.8	91.5	97.5
		1000	10	100	99.3	91.7	89.4	91.3	97
		1000	15	100	100	97.5	96.9	99.4	98.2
		2000	5	97.1	46.1	63.1	43.1	60.6	91.3
		2000	10	98.4	57.7	66.04	59.6	64.2	92
		2000	15	98.8	78.1	48	36.5	39	90.7
		3000	5	97.2	57.5	64.6	51.5	51.5	89.6
		3000	10	96.9	51	57.1	52.7	52.7	84.6
		3000	15	99	62.2	43.96	37.7	39.2	85
2000	5	1000	5	98.1	62.8	45.8	49	53.8	88.3
		1000	10	96.8	56.4	51.5	35.8	42.3	91.8
		1000	15	97.1	48.6	50.6	39.4	49.8	88.1
		2000	5	100	100	97.1	98.3	99.6	99.6
		2000	10	100	100	84.5	86.4	85.1	99.2
		2000	15	99.4	99.3	86.87	87.1	87.8	98.8
		3000	5	99.4	74	72.3	73.6	72	97.6
		3000	10	98.1	70	65.6	65.6	66.9	95.7
		3000	15	97.6	70	68.3	66.7	68	96.3
2000	10	1000	5	97.4	67	43.1	31.4	32.7	86
		1000	10	98.6	69.27	45	39	31.9	94.6
		1000	15	98.1	51.7	42.3	36.2	30	83.9
		2000	5	100	98.2	83.2	81.2	80.3	93.4
		2000	10	100	100	99.6	100	100	100
		2000	15	99.6	98.4	86	88.3	87.5	98.1
		3000	5	99	66.74	70	71.3	74.3	94.6
		3000	10	99.2	72.6	68.5	71.9	69	85.7
		3000	15	98	70.8	69.4	70.1	68.9	88.8
2000	15	1000	5	97.3	73.4	51.3	51.2	45.6	81.2
		1000	10	96.8	73.4	47.3	45.1	43.8	82.5
		1000	15	99.1	77.5	53.3	49.5	43	83.2
		2000	5	99.3	98.4	87.9	84	82	95.8
		2000	10	99.3	99.3	90.62	85.8	81	96.4
		2000	15	100	100	98.5	99.2	99.8	100
		3000	5	94.7	46.7	71.3	64.2	77.1	86.3
		3000	10	97	49.8	61.3	64.2	71.5	90.3
		3000	15	98.1	53.9	73.2	79.2	79.8	85.7
3000	5	1000	5	98.4	63.57	41.3	24.2	27.1	89.3
		1000	10	95.4	55.3	45.8	40.8	31	89.6
		1000	15	97.2	55.4	36.3	37.3	25.4	85.4
		2000	5	98.9	74	71.2	74.8	58.3	84.2
		2000	10	98.1	68.85	66.7	67.9	77.3	97.6
		2000	15	95.5	46.25	71.7	75.4	75.8	91.3
		3000	5	100	100	99	99	99.8	100
		3000	10	99.8	100	87.3	87.4	86.2	99.3
		3000	15	100	100	88.2	87.8	87.3	99.6
3000	10	1000	5	96.1	57.83	26.2	19.4	14	85.7
		1000	10	97.3	50	35.8	12.3	10.6	82.1
		1000	15	95	28.5	23.5	16	11.25	86.1
		2000	5	98.5	69.1	60.8	56.9	53.3	87.6
		2000	10	99.1	72.3	62.7	67.8	66	96.9
		2000	15	97.1	51.6	61.5	66.5	58.3	85.5
		3000	5	99.6	98.2	85.6	82.9	83.8	99.3
		3000	10	100	100	94.6	92.5	99.4	100
		3000	15	100	99.6	84.4	89	85.6	100
3000	15	1000	5	97.1	38.8	22.5	27.3	30.4	83
		1000	10	96.9	38.9	35.8	33.5	33.1	81.1
		1000	15	99.1	48.9	29.4	31.7	29.8	84.3
		2000	5	97.6	73.2	66.9	65	63.5	89.2
		2000	10	98.9	72.8	73.1	77.3	78.7	98.5
		2000	15	98.9	75.8	74	75.6	75.4	85.3
		3000	5	99.2	100	90.6	89.5	82.8	99.8
		3000	10	100	100	83.6	84.7	86.3	100
		3000	15	100	100	94.2	95.8	99.4	100
Overall Average Prediction Accuracy				98.4	73.7	67.4	64.7	65.3	92.3

**Table 6:** Performance of the proposed methodology in terms of prediction accuracy for defect diagnosis under variable speed (case study-2).

Training	Testing	Prediction Accuracy (%)	
		Ball Bearing	Cylindrical Roller Bearing
3170 rpm (Constant Speed)	3170 rpm (Constant speed)	100	100
	4955 rpm (Constant speed)	100	99.9
	1000–2000 rpm (acceleration)	99.1	97.2
	3525–4125 rpm (acceleration)	99.4	96.8
	4050–2560 rpm (deceleration)	99.1	96.2
1000–2000 rpm (acceleration)	3170 rpm (Constant speed)	98.7	99.1
	4955 rpm (Constant speed)	98.5	98.1
	1000–2000 rpm (acceleration)	100	100
	3525–4125 rpm (acceleration)	99.4	97.3
	4050–2560 rpm (deceleration)	98	95.1
Average Prediction accuracy for each bearing		99.2	98
Overall average Prediction accuracy		98.6	

## Data Availability

Not applicable.
